# Chemical Characterization, Gastrointestinal Motility and Sensory Evaluation of Dark Chocolate: A Nutraceutical Boosting Consumers’ Health

**DOI:** 10.3390/nu12040939

**Published:** 2020-03-28

**Authors:** Giusy Rita Caponio, Michele Pio Lorusso, Giovanni Trifone Sorrenti, Vincenzo Marcotrigiano, Graziana Difonzo, Elisabetta De Angelis, Rocco Guagnano, Agostino Di Ciaula, Giusy Diella, Antonio Francesco Logrieco, Maria Teresa Montagna, Linda Monaci, Maria De Angelis, Piero Portincasa

**Affiliations:** 1 Department of Soil, Plant and Food Sciences, University of Bari Aldo Moro, via Amendola 165/a, 70126 Bari, Italy; giusy.caponio@uniba.it (G.R.C.); maria.deangelis@uniba.it (M.D.A.); 2 Clinica Medica “A. Murri”, Department of Biomedical Sciences & Human Oncology, University of Bari Medical School, Piazza G. Cesare 11, 70124 Bari, Italy; 3 Food Hygiene and Nutrition Service, Department of Prevention, Local Health Unit BT, 76125 Barletta-Andria-Trani, Italy; sorrentigianni@tiscali.it (G.T.S.); vincenzo.marcotrigiano@aslbat.it (V.M.); 4 Institute of Sciences of Food Production, National Research Council of Italy (ISPA-CNR), Via Amendola 122/o, 70126 Bari, Italy; elisabetta.deangelis@ispa.cnr.it (E.D.A.); antonio.logrieco@ispa.cnr.it (A.F.L.); 5 Department of Biomedical Sciences and Human Oncology, Section of Hygiene, University of Bari “Aldo Moro” Medical School, Piazza G. Cesare 11, 70124 Bari, Italymariateresa.montagna@uniba.it (M.T.M.)

**Keywords:** polyphenols, high resolution mass spectrometry, orocecal transit time, ultrasonography, chemical characterization, chocolate, nutraceutical

## Abstract

We performed a comprehensive study encompassing chemical characterization and sensory evaluation of two types of dark chocolate, i.e., artisanal (Choco-A) and industrial (Choco-I), as well as an evaluation of onset of gastrointestinal symptoms and gastrointestinal motility in healthy subjects fed with dark chocolate. Proteomic, lipid and metabolite analysis were performed by LC-MS/MS analysis and the total phenol content and antioxidant activity were estimated in both types of chocolate. Fifty healthy volunteers joined the study of the sensory characteristics of both types of chocolate; another 16 subjects underwent the study of gallbladder and gastric emptying by functional ultrasonography and orocecal transit time by lactulose H_2_-breath test after ingestion of dark chocolate. Identification of polyphenols, amino acids and fatty acids was carried out in both types of chocolate analysed, and results confirmed their richness in polyphenols, amino acid derivatives and fatty acids (FAs) either saturated (stearic, myristic, palmitic, ecosanoic) or unsaturated (oleic and linolenic). For agreeability, Choco-A scored higher than Choco-I for smell, texture, and taste and they did not show significant differences in the gastrointestinal motility. In conclusion as for gastrointestinal motility studies, we report that the ingestion of a small amount of chocolate induced a mild gallbladder, gastric contraction and a fast transit time compared to the test meal in healthy subjects.

## 1. Introduction

In recent decades, nutrition research has focused on the investigation of bioactive dietary compounds widely found in many plant-based foods and beverages, with the aim of elucidating their beneficial properties to human health. Cocoa (*Theobroma cacao* L.) and chocolate products appear to be one of the most promising foods for their high polyphenol and alkaloid contents [1]. Cocoa and chocolate have a pleasant taste and beneficial effects have been described on health, mediated by lipid and antioxidant components. Dark chocolate consists of about 43% lipid, mainly cocoa butter (as 33% oleic acid, 25% palmitic acid and 33% stearic acid) [2,3]. Chocolate is a renowned source of flavanols such as epicatechin, catechin, and procyanidins that may offer cardiometabolic protection through several mechanisms, including antihypertensive, antiplatelet, antioxidant, and anti-inflammatory effects [4,5]. A meta-analysis study put in correlation chocolate intake and the risk of coronary heart disease (CHD), stroke, and diabetes, concluding that consuming chocolate in moderation (≤6 servings/week) can be optimal for preventing these disorders. Other bioactives in chocolate, in particular methylxanthines, enhance the effects of cocoa flavanols on cardiovascular function [6]. In addition, flavanols in chocolate have been reported to improve insulin sensitivity, by promoting the survival and function of pancreatic β-cells and by improving the insulin signaling pathway in hepatic cells [7]. Dark chocolate ingestion and cocoa polyphenol content was reported to modulate intestinal microbiota, thus leading to the growth of bacteria that triggered a tolerogenic anti-inflammatory pathway in the host. In a human trial conducted on healthy volunteers, consumption of a high-cocoa flavanol beverage for four weeks proved to significantly increase the growth of *Lactobacillus* spp. and *Bifidobacterium* spp., bacteria capable of maintaining an anti-inflammatory status in the bowel, in comparison to a low cocoa flavanol drink [8]. This study suggests that cocoa polyphenols may behave as prebiotics and trigger a tolerogenic pathway in the gut. In addition, the consumption of dark chocolate can influence basal human metabolism, as reported by Francois-Pierre J. Martin et al. They studied the reduction of cortisol and catecholamine production as a result of chocolate consumption on a group of 30 human subjects, with low and high anxiety traits [9].

Based on these considerations, we focused on dark chocolate, deemed a nutraceutical food. For the current study two representative types of dark chocolate (70% of cocoa), artisanal and industrial, were chosen in order to investigate eventual differences and if they might exert any influence on the clinical tests. In the present paper, we chemically characterized both types of chocolate and tested the organoleptic perceptions in a restricted group of consumers identified. Because of the distinctive gut response to food components [10], we also studied the gastrointestinal motility patterns (i.e., stomach and gallbladder by ultrasonography, and orocecal transit by hydrogen breath test) in response to the consumption of both types of dark chocolate, as compared to a standard test meal.

In this pilot study, we enrolled 50 subjects for the sensory evaluation of both types of chocolate, in addition to a subgroup of 16 individuals assessed for the motility studies of the gastrointestinal tract. The well-balanced group of subjects consisted of healthy volunteers who provided data to build up a reference database about perception and motility parameters, thus avoiding any interference due to other factors, such as old age, overweight, obesity, other diseases, and symptoms.

The choice of using the standard test meal in the present work was based on our previous extensive experience dealing with groups of healthy subjects and different types of patients, as already reported [11].

In this comprehensive study, we therefore combined chemical analyses, organoleptic evaluation, and gastrointestinal motility studies of two types of dark chocolate, while providing novel and physiologically relevant updates.

## 2. Materials and Methods 

**Figure 1:**
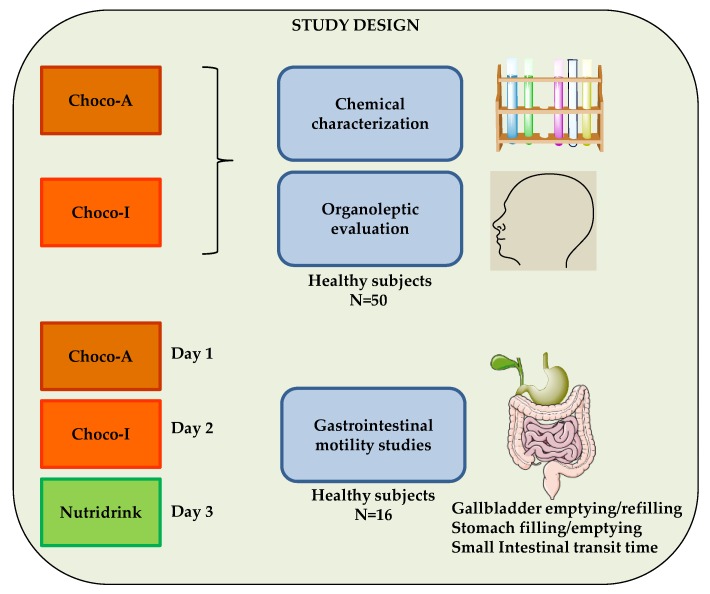
The diagram is shows the study design. Chemical analyses, organoleptic evaluation and clinical study of two dark chocolates compared with a standard meal (Nutridrink). Choco-A, artisanal; Choco-I, industrial.

The study design consisted of chemical characterization of two types of chocolate, Choco-A, artisanal and Choco-I, industrial. In addition, organoleptic evaluation of both chocolates was performed by 50 healthy subjects enrolled in the study. The workflow also included a clinical study: specifically, gastrointestinal motility studies of the two types of dark chocolate and a standard meal (Nutridrink), in three different days, on a subgroup of 16 healthy subjects (Figure 1).

### 2.1. Chemical Characterization of Chocolate Samples

Chemical analyses of dark chocolates encompassed determination of the soluble fibre content, the total phenol content determination, antioxidant activity evaluation and polyphenols quantitation by HPLC-DAD. Characterization of chocolate proteins as well as metabolite profiling and analysis of fatty acids were performed by LC-MS/MS analysis.

#### 2.1.1. Chemicals 

Antioxidant and polyphenols analysis: DPPH (2,2-diphenyl-1-picrylhydrazyl), ABTS (2,2-Azino-bis (3-ethylbenzothiazoline-6-sulphonic acid), Folin-Ciocalteu reagent, (-)-Epicatechin, Theobromine and Procyanidin B2 were obtained from Sigma-Aldrich (Milan, Italy); Formic acid (for HPLC) was purchased from Fluka (Milan, Italy). Acetonitrile (for HPLC), Acetic acid (for HPLC) and Ethanol were purchased from Carlo Erba Reagents (Cornaredo, Milan, Italy). Acetone was provided by VWR International S.r.l. (Milan, Italy).

Proteomic analysis: Trizma-base and Urea were obtained from VWR International S.r.l. (Milan, Italy), while Exane (for HPLC), ammonium bicarbonate (AMBIC), iodoacetamide (IAA) and dithiothreitol (DTT) were provided by Sigma Aldrich (Milan, Italy). Acetonitrile (Gold HPLC ultragradient) were purchased from Carlo Erba Reagents (Cornaredo, Milan, Italia) while ultrapure water used was produced by a Millipore Milli-Q system (Millipore, Bedford, MA, USA). Formic acid (MS grade) was purchased from Fluka (Milan, Italy) whilst syringe filters (0.45 µm of porosity in regenerated cellulose RC, and 5 µm of porosity in cellulose acetate CA) were purchased from Sartorius (Gottingem, Germania). Trypsin (proteomic grade) for protein digestion was purchased from Promega (Milan, Italy).

Lipidomic investigation: Methanol (HPLC grade), Cholorophorm (HPLC grade) and ammonium acetate were obtained from VWR International S.r.l. (Milan, Italy) while 0.45 µm syringe filters in Polytetrafluoroethylene (PTFE) were purchased from Sartorius (Gottingen, Germania). Acetonitrile (Gold HPLC ultragradient) were purchased from Carlo Erba Reagents (Cornaredo, Milan, Italy).

Chemical compounds analysis: Formic acid (for HPLC) was purchased from Fluka (Milan, Italy). Acetonitrile (for HPLC), Acetic acid (for HPLC) and Ethanol were purchased from Carlo Erba Reagents (Cornaredo, Milan, Italy). Acetone was provided by VWR International S.r.l. (Milan, Italy).

#### 2.1.2. Chocolate Samples

Two type of dark chocolate containing 70% of cocoa, namely artisanal (Choco-A) and industrial (Choco-I), in two different production batches, were purchased from a local market and each analyzed in duplicate (total of eight samples). Each single chocolate bar (75 g) was weighed in a mixer bowl (cooled at +4 °C) and grinded in a commercial blender (Sterilmixer 12 model 6805-50; PBI International) according to the following procedure: i) mix 3 sec at full speed, stop and wait 3 sec; ii) mix 3 sec at medium speed, stop and wait 3 sec; iii) mix 3 sec at low speed, stop and wait 3 sec. After each step the bowl and its content were cooled for 2 min at +4 °C. Each step (i-iii) was repeated once more. Minced chocolate was finally passed through a 1 mm sieve and stored at +4 °C.

#### 2.1.3. Proximate Composition

The protein content (total nitrogen × 5.7), ashes, and moisture content were determined according to the AOAC methods 979.09, 923.03 and 925.10, respectively [12]. The soluble fibre content determination was performed with AOAC 993.19 1996 method [13]. The lipid content was determined by means of a Soxhlet apparatus using diethyl ether (Sigma-Aldrich Chemical Co., St. Louis, MO, USA) as extracting solvent, as described in AOAC Official Method 945.38F [14]. The carbohydrate content was calculated by subtraction. 

### 2.2. Total Phenol Content Determination and Antioxidant Activity Evaluation

#### 2.2.1. Sample Preparation

The extraction from the chocolate samples was performed by solid-liquid extraction using ethanol as the solvent, following the procedure reported in Gu et al. 2006 [15] with modifications. Chocolate (1 g) was washed with hexane (ratio 1:5 w/v) to remove the lipidic phase and with ethanol 80% v/v in a ratio 1:10 w/v. After an intense agitation with vortex at 10,976 g/min for 10 min at room temperature, a sonication (C.E.I.A S.p.A., CP 104, Viciomaggio, Italy) for 15 min was performed, followed by centrifugation (Thermo Fisher Scientific, Osterodeam Harz, Germany) at 15,805 g/min for 10 min. Three washes were performed for each sample. The hydroalcoholic extracts were filtered through filter papers (Cordenons, PN, Italy) and nylon filters (pore size 0.45 mm, Sigma, Ireland). 

#### 2.2.2. Total Phenol Content Determination

The determination of total phenol content (TPC) was performed by Folin-Ciocalteu method according to Difonzo et al. (2019) [16]. 20 μL of chocolate extract was added to 980 μL of ddH_2_O and 100 µL of Folin-Ciocalteu reagent. After 3 min, 800 µL of 7.5% Na_2_CO_3_ solution was added, following incubation at room temperature for 60 min. The absorbance was read at 720 nm using a Cary 60 spectrophotometer Agilent (Cernusco, Milan Italy). The TPC was expressed as mg gallic acid equivalents (GAE)/g of chocolate. The antioxidant activity determination was carried out by DPPH and ABTS assays reported in Difonzo et al. [17]. The DPPH assay was performed by preparing a solution of DPPH 0.08 mM in ethanol. In cuvettes for spectrophotometry, 50 µL of each sample were added to 950 µL of DPPH solution. After 30 min in the dark, the decrease of absorbance was measured at 517 nm using a Cary 60 spectrophotometer Agilent (Cernusco, Milan, Italy). The ABTS radical was generated by chemical reaction with potassium persulfate (K_2_S_2_O_8_). For this purpose, 25 mL of ABTS (7 mM in H_2_O) was spiked with 440 μL of K_2_S_2_O_8_ (140 mM) and kept in the dark at room temperature for 12–16 h. The working solution was prepared by diluting with H_2_O to obtain a final absorbance at 734 nm equal to 0.80 ± 0.02 [18]. The decrease of absorbance was measured at 734 nm after 8 min of incubation. The results were expressed as μmol Trolox equivalents (TE)/g of chocolate. Each sample was analyzed in triplicate.

### 2.3. Polyphenol Investigation by LC/High Resolution Mass Spectrometry

#### 2.3.1. Sample Preparation

As first step LC-MS/MS analysis was accomplished on chocolate extracts with the aim to identify the main polyphenols characterizing both types of chocolate (artisanal and industrial). The extraction procedure was performed according to Ortega et al. [19]. Briefly, 1 g of minced chocolate was defatted with 5 mL of hexane to get the final ratio of 1:5 v/v. Hexane was then removed and the defatted chocolate powder was combined with the solvent extraction (acetone/MilliQ water/acetic acid, 70/29.5/0.5, v/v/v) at the final ratio of 1:10. After a vigorous vortexing for 10 min (10,976 g min^−1^) at room temperature, the extract was sonicated in ultrasound bath (C.E.I.A S.p.A., CP 104, Viciomaggio, Italy) at room temperature for 15 min and finally centrifuged (Thermo Fisher Scientific, Osterodeam Harz, Germany) at 15,805 g/min for 10 min. The supernatant was collected and stored in a clean vial. The remaining pellet was then submitted to additional two extraction cycles (making a total of three extraction cycles). The supernatants obtained from each extraction step were then pooled together and filtered on filter papers (Cordenons, PN, Italy). The organic solvent was removed by rotary evaporation (Buchi, Labortechnick AG, Switzerland) under partial vacuum. Finally, the water extract was freeze-dried (Hetossic freeze drier A. De Mori CD-13-2, Milan, Italy) to obtain the dried phenolic extract that was stored at −20 °C before analysis.

#### 2.3.2. LC-MS/MS Analysis

Polyphenols identification experiments were accomplished on a Q-Exactive™ Plus Hybrid Quadrupole-Orbitrap™ High Resolution Mass Spectrometer coupled to a UHPLC pump system (Thermo Fisher Scientific, Bremen, Germany). Chromatographic separation of polyphenols was carried out on an Acclaim 120-Dionex Bonded Silica C18 column (3 µm, 120 Å, 3 × 1450 mm, Thermo Fisher Scientific, Bremen) at a flow rate of 250 µl/mL according to the following elution gradient: 1 min of isocratic condition at 5% solvent B. From 1–40 min, solvent B increased from 5% to 60%, there was a further increase between 40–41 from 60% to 95%, then kept constant for 5 min. In 1 min, solvent B decreased to 5% remaining constant for 20 min for column conditioning before next injection. Solvent A = H2O + 0.1% FA; solvent B= Methanol + 0.1% FA. The temperature of the column was kept to 35 °C along the run, the volume injection was set to 20 µL and each sample was injected twice in MS. Spectra were acquired both in positive and negative ion mode in the mass range of 120–1200 m/z by operating in FullMS/All Ion Fragmentation (FullMS/AIF) acquisition mode. Mass resolution was set to 140,000 FWHM.

### 2.4. Polyphenols and Theobromine Quantitation by HPLC-DAD 

Selected polyphenols compounds, namely catechin, epicatechin and procyanidin B2, along with the alkaloid theobromine identified in the chocolate extracts previously analyzed, (see par. 2.1.5) were finally analysed by HPLC-DAD for quantification purpose. The HPLC-DAD analysis was conducted with a Thermo Scientific UHPLC system (Dionex, Germering, Germany) equipped with an auto-sampler, a quaternary pump, a vacuum degasser, a diode array detector, and a thermostatic column compartment. The separation was carried out with an analytical column RP-C18 column AcclaimTM 120-Thermo Fisher, 3 µm particle size, 120 Å pore size, 150 × 3.0 mm operated at 35 °C. The mobile phases were solvents as follows: solvent A = water/formic acid (99/1, v/v) and solvent B = methanol/formic acid 127 (99/1, v/v). The elution program was (min; %B): (0;2) (1;2) (6.5;15) (9;15) (12;30) (14;30) 128 (27;75) (32;95) (37;95) (40;2) (45;2). The flow rate was 0.800 mL min^−1^ with an injection volume set at 20 μL. UV detection was set at 240, 280, 320 and 520 nm. A stock standard solution of 1 mg/mL of (-)-epicatechin, ( + )-catechin, procyanidin B2 and caffeine was dissolved in acetonitrile. Theobromine was dissolved in acetone/MilliQ water/ acetic acid (70/29.5/0.5, v/v/v).

### 2.5. Chemical Profiling of Chocolate 

Chemical profiling (artisanal and industrial) was carried out by LC-MS/MS analysis on a Q-Exactive™ Plus Hybrid Quadrupole-Orbitrap™ High Resolution Mass Spectrometer coupled to a UHPLC pump system (Thermo Fisher Scientific, Bremen, Germany). Experiments were accomplished on the same chocolate extracts used for polyphenols identification as already detailed in the previous section. Separation was accomplished by applying the same conditions reported in par. 2.1.4. MS spectra were acquired both in positive and negative polarity by setting the instrument in FullMS/Data dependent acquisition mode (FullMS/DD2). For FullMS and dd-MS2 scan event, resolution was set to 70,000 FWHM and 17,500 FWHM, respectively while dd-setting maximum AGC target value was set at 5.00 e1 and ions with charge higher than 4 were excluded.

Raw MS spectra were then processed via the commercial software Compound Discoverer v.3 (Thermo Fisher Scientific) specific for metabolite profile and identification. 

Some constraints were applied in order to increase confidence in the identification and the following criteria were set: intensity not less than 300,000 and S/*n* larger than 3 in each set of data that were extracted and merged into components according to selected ion adducts.

Unknown compound identification and prediction of the elemental composition was accomplished by activating the ChemSpider and mzCloud nodes search. Mass tolerance set for the search was better than 3 ppm. All compounds identified by software were finally visually inspected by the analyst and only those fulfilling the conditions for identification by ChemSpider and mzCloud (with the main respective fragments) were taken into account. 

### 2.6. Lipid Analysis (or Analysis of Fatty Acids) by LC-MS/MS 

#### 2.6.1. Sample Preparation

Lipid fraction was extracted in chocolate samples by applying the Bligh & Dyer protocol [20]. Briefly, 1 g of sample was mixed with 2 mL of chloroform and 4 mL of methanol followed by shaking for 2 min. Then, 2 mL of fresh chloroform was added and the final mixture was vortexed for 2 min. Finally, 3.6 mL of water was added to the mixture and, after vigorous shaking for 2 min, it was centrifuged at 771 g for 10 min. The lower phase was collected and transferred to a clean vial. The remaining pellet was then submitted to a second extraction step by adding 4 mL of a mixture of chloroform/methanol (90:10 v/v) followed by a 1 min shaking and a final centrifugation for 10 min at 771 g. The lower phase was then collected and mixed to the first extract. Finally, 1 mL of lipid extract was dried and dissolved in 2 mL of chloroform-methanol (1:1v/v), filtered through a 0.45 µm PTFE syringe filter and analyzed by LC-MS/MS.

#### 2.6.2. LC-MS/MS Analysis

Lipid fraction was analyzed by LC-MS/MS equipment consisting of a Q-Exactive™ Plus Hybrid Quadrupole-Orbitrap™ High Resolution Mass Spectrometer coupled to a UHPLC pump system (Thermo Fisher Scientific, Bremen, Germany). The mixture of lipids (5 µL injection volume, *n* = 2 replicates) was chromatographically separated on an Acclaim Mixed Mode HILIC-1 (150 × 2.1 mm, 3 µm, 120 Å, Thermo Fisher Scientific) as follows: 0–6 min isocratic at 90% of solvent B, 6–15 min gradual decrease of solvent B down to 60%, 15–25 min constant at 60% of B, 25–30 min gradual increase of solvent B up to 90%. These conditions were kept constant for 10 min for column conditioning. The mobile phases used for chromatographic separation were: A: MeOH:H_2_O 90:10 (v/v) + 1 mM Ammonium acetate; B: AcN:H_2_O 90:10 (v/v) + 1 mM Ammonium acetate. The flow rate was set to 300 µl/min and the temperature of the column set at 25 °C. MS analysis was performed both in positive and negative polarity by running the instrument in FullMS/Data dependent acquisition mode (FullMS/DD2). Resolution was set to 70,000 FWHM and 35,000 FWHM for FullMS and dd-MS2 scan event respectively, while dd-setting maximum AGC target value was set at 6.00e3.

Lipid identification was performed by processing raw MS data with the software Compound Discoverer 3.0 (Thermo Fisher Scientific, Bremen Germany) by setting the same parameters as detailed in par. 2.1.6. with exception for ChemSpider node where the only database interrogated was LipidMaps. Software results were filtered according to the same criteria above reported (par. 2.1.6). 

### 2.7. Protein Characterization by HPLC-MS/MS Analysis

The chemical profiling of both chocolates was finally extended also to the protein fraction. Protein identification was achieved by bottom-up proteomics, where proteins are identified by analyzing the peptide mixture generated upon digestion operated by proteolytic enzyme with specific cleavage sites. 

#### 2.7.1. Sample Preparation

Chocolate samples were firstly defatted by adding 50 mL of hexane to 5 g of chocolate powder [ratio 1:10]. The mixture was left under shaking (250 rpm) for 1 h at room temperature and after a 30 min decantation the hexane phase was removed and discarded. Defatting step was repeated twice. The remaining powder was left drying under a gentle stream of air and finally weighted. Protein extraction was accomplished by adding 7 mL of 200 mM TRIS- HCl pH 9.2, 5 M urea extraction buffer to 0.7 g of defatted chocolate powder (ratio of 1:10) followed by vortexing for 2 min, shaking for 30 min at 250 rpm (room temperature) and sonication in ultrasound bath (Sonomatic, Langford Ultrasonics, Birmingham B30 2HY, England) for 15 min. Sample was then centrifuged for 15 min at 3900 g at 18 ° C and the supernatant collected and filtered through a 5 µm Cellulose Acetate filter. These extracts were quantified using the BCA test using the indications in the kit (ABP Biosciences, Beltsville, MD). For protein digestion, chocolate extract was firstly diluted 50 times with 50 mM Ammonium Bicarbonate (AB) buffer and an aliquot of 300 µL denatured at 95 °C for 15 min and submitted to enzymatic digestion according to the protocol by Pilolli et al. [21]. The final extract was centrifuged at 9500 g for 10 min and filtered on 0.45 µm RC filters before injection. 

#### 2.7.2. LC-MS/MS Analysis and Database Search

For HPLC–MS analyses a system composed of a UHPLC pump provided with an autosampler and ESI interface coupled to a high resolution mass spectrometer (Q-Exactive™ Plus Hybrid Quadrupole-Orbitrap™ High Resolution Mass Spectrometer, Thermo Fisher Scientific, Bremen) was employed. A peptides mixture (20 µL injection volume, *n* = 2 replicates) was separated on a reversed phase Aeris peptide analytical column (internal diameter 2.1 mm, length 150 mm, particle size 3.6 μm, porosity 100 Å, Phenomenex, Torrance, CA, US) at a flow rate of 200 μL/min. The chromatographic gradient used was the following: 0–1 min isocratic at 5% of solvent B; 1–40 min gradual increase of solvent B up to 60%; 40–41 min increase of solvent B up to 95% kept constant for 5 min to allow column washing; from 46–48 min decrease of solvent B to 5% B kept constant for 15 min for column conditioning. The mobile phases used for chromatographic separation were A: H_2_O + 0.1% Formic Acid, B: Methanol + 0.1% Formic Acid and the temperature of the column during the chromatographic run was set to 25 °C.

MS spectra were acquired in positive ion mode by running the instrument in Full-MS/dd-MS2 acquisition mode, setting the resolution to 70000 and 17500 FWHM for FullMS and ddMS2 event, respectively. Raw spectra were finally processed using the Proteome Discoverer software 2.1.1.21 (Thermo Fisher Scientific, Bremen Germany) and protein identification was achieved by SequestHT search against the cocoa database extracted by Swiss Prot DB basing on the taxonomy code of *Theobroma cacao* (Uniprot ID: 3641). The identification of tryptic peptides originated by digestion experiments was accomplished by setting at 5 ppm and 0.05 Da, respectively, the mass tolerance on the precursor and fragment ions. Only trustful peptide-spectrum matches were accepted and in particular a minimum of two peptides or higher were the minimum criteria for protein identification by selecting a high (FDR < 1%) and medium confidence (FDR < 5%).

## 3. Clinical Study

Overall, 50 healthy volunteers participated in the sensory evaluation test, while another 16 subjects underwent the gastrointestinal motility studies (Figure 1). Volunteers were Italian, in particular local residents, nurses, medical staff, and students willing to participate, and signed the informed consent. Diseases or ongoing/previous medications were absent in the subjects enrolled in the study in order to avoid adding confusing factors to the perception and motility studies. The study was advertised internally (intra-hospital presentations), since no public, media advertisement or payment is allowed in Italy, according to ethical regulations. In the case of the sensory evaluation study, for a *t*-test, *n* = 33 was the minimum sample size needed to obtain a power of 0.80, when the effect size is medium and a significance level of 0.05 is employed (calculated with R software, package “pwr”). For this reason, we enrolled a larger number of volunteers (*n* = 50) in order to avoid subsequent sample bias due to possible drop-outs. We selected a homogeneous gender and age-balanced healthy group of adults for building a solid database concerning individual perception about both chocolates. The subgroup of 16 participants selected for further investigations gave their consent to undergo the gastrointestinal motility studies which consisted of three different tests over three different days, for an average duration of no less than 3 hrs (overall).

The clinical characteristics of the enrolled subjects appear in Table 1. The proportion of males and females was similar in both groups. Exclusion criteria were diagnosis of organic diseases, therapies potentially influencing sensory perception or gastrointestinal motility, and allergy to chocolate or chocolate components. All subjects gave their informed consent and, at entry, underwent a full clinical evaluation in order to exclude clinically evident diseases or drug consumption, two conditions potentially able to act as confounder factors. The study was no-profit and approved by the Ethics Review Board of the University Hospital Policlinico in Bari (study number: 5921/ prot. n° 0059322|09/07/2019 |AOUCPG23|COMET|P).

### 3.1. Organoleptic Evaluation

**Figure 2:**
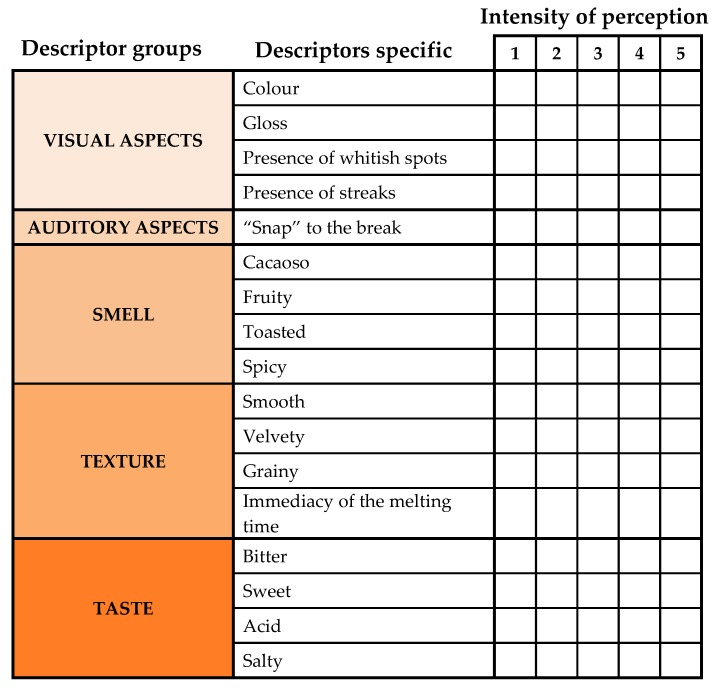
Forms employed for the organoleptic evaluation of the chocolates.

Subjects underwent the organoleptic assessment of the two dark chocolates in blind mode. Perceptions were recorded using either quantitative visual analogue scales (VAS, i.e., a 0–100 mm horizontal line, 0 = absent and 100 = maximum, drawing a mark at the point of perception) and semi-quantitative scales (score 1–5). Subjects had to record the degree of several domains referred to as visual (colour, gloss, presence of whitish spots, presence of streaks); auditory (“snap” of the breaking chocolate); smell (chocolaty, fruity, toasted and spicy); texture (smooth, velvety, grainy, immediacy of the melting time); and taste (bitter, sweet, acid, salty) (Figure 2) [22]. Each chocolate test required a total of two hours, and the subjects during chocolate tasting were asked to wash their mouth with plain water to avoid any potential influence that might impair the flavour. Other food, drinks, smoking and physical activity were avoided before and during the test.

### 3.2. Gastrointestinal Motility Studies

The simultaneous dynamic, time-dependent response of the stomach (emptying), gallbladder (emptying), and small intestine (orocecal transit time (OCTT)/fermentation) were measured in 16 healthy subjects on three different days with Choco-A, Choco-I, and a standard test meal, whose composition appears in Table 2. In order to standardize the gastrointestinal response, each test meal consisted of a final solid/liquid volume of 215 mL, namely:

- Choco-A: chocolate 28.6 g + water 175 mL + Lactulose 15 mL;
- Choco-I: chocolate 27.3 g of + water 176 mL + Lactulose 15 mL;
- Nu: Nutridrink^®^ (Nutricia, Milano, Italy), 200 mL + Lactulose 15 mL.

To induce intestinal fermentation, 15 mL of lactulose (Lattulac^®^, SOFAR, Trezzano Rosa, Milan, Italy) was added to each meal. 

Thus, the test meals (Chocolates and Nutridrink) were different in composition but isovolumetric, isodense and isolipidic. The calories of Nutridrink were almost twice those of both chocolates. 

Experiments were planned randomly on three different days, starting at 8 am after an overnight fast of at least 12 h. The meal was ingested at room temperature in 1 min in the presence of the examiner.

Gallbladder, gastric motility and orocecal transit time were studied simultaneously by ultrasonography and H_2_-breath test, as previously reported by our group [11,23,24,25,26]. Time-dependent changes of fasting and postprandial gallbladder volumes (mL) and antral areas (cm²) were measured from frozen sonograms on a portable scanner (Noblus, Hitachi Medical, Tokyo, Japan) equipped with a 3.5 MHz convex transducer. 

Gallbladder volume was measured at the level of the right hypochondrium, assuming an ellipsoid shape of the organ, using the length, width, and depth of the viscus [27,28]. Antral area was measured at the level of the epigastrium. Measurements were recorded at: 0 min (baseline), every 5 min until 30 min, and then every 15 min up to 120 min. Indices of gallbladder emptying were fasting volume (mL), residual volume (minimum volume measured postprandially, in mL and percentage of fasting volume), time to residual volume (min), final residual volume (mL and % of fasting volume), area-under-emptying curve (AUC as mL x 120 min and as % x 120 min), and half-refilling time (min). Indices of gastric emptying were basal antral area (cm^2^), maximal postprandial antral area recorded 5 min after meal ingestion (time 0), and postprandial and minimal postprandial antral areas during the 2 h emptying curve. Postprandial areas were also normalized to maximal areas after subtraction of basal areas, i.e., 100 × (At - Abas)/(Amax - Abas), where At = postprandial area at any given time; Abas = basal area; Amax = maximal antral area. The AUC was expressed as mL and % × 120 min, and half-emptying time (T1/2, min). T1/2 was calculated by linear regression analysis from the linear part of the emptying curves and was the time at which 50% decrease of gallbladder volume and antral area occurred.

OCTT was measured by the lactulose H_2_-breath test according to standard guidelines [25,29,30,31]. During the 10 days before the test, antibiotics, probiotics, or other drugs known to affect gastrointestinal motility or intestinal microbiota were prohibited. A special diet was given the day before the test, to avoid the presence of non-absorbable or slowly fermentable food in the intestinal tract. The diet consisted of meat, fish, eggs and olive oil, and water as drink. Breath samples were taken before the meal and, subsequently, every 10 min up to 120 min after the ingestion of meal, during which a rise of 10 p.p.m. above baseline on two consecutive measurements (i.e., OCTT in min) was observed in all subjects. Time-dependent changes of H_2_ in expired breath were studied using a pre-calibrated, portable hydrogen-sensitive electrochemical device (EC60Gastrolyzer; Bedfont Scientific, Medford, NJ, USA). Results are expressed as H_2_ excretion in parts per million (p.p.m.). Accuracy of the detector was ±2 p.p.m.

## 4. Statistical Analysis 

Analyses were performed using the statistical software NCSS10 (NCSS LLC, Kaysville, UT, USA). Values are mean ± standard error of the mean (SEM); differences between two groups, concerning sensory evaluation, were evaluated by the Student’s t-test. Differences between the indices of motility were checked by the analysis of variance (ANOVA) followed by Tukey-Kramer’s all pairs simultaneous confidence intervals of mean difference. Results were considered significant at the 5% critical level (P < 0.05).

## 5. Results

### 5.1. Chemical Analyses

Cacao trees belong to the *Malvaceae* family. Cocoa beans are extracted from the cocoa fruit pod of cacao trees (*Theobroma cacao*). They are highly abundant in carbohydrates, fat, and protein. On the other hand, they represent also a source of vitamins, minerals, and polyphenols, consequently claiming the definition of nutraceutical. It is well known that cocoa beans yield chocolate via a process involving fermentation, roasting, and grinding. The proximate composition of Choco-A and Choco-I (Table 3) shows similar results in terms of carbohydrates percent, soluble fiber, fat, protein, moisture, ash, and total phenol content. 

In addition, chemical analyses showed that chocolates were similar in terms of total phenol content (Folin-Ciocalteu test), antioxidant activity and DPPH assay, main phenolic compounds including theobromine, catechin, procyanidin B2, and epicatechin (Table 4).

Among the contained proteins, the 2S cocoa seed albumin storage polypeptide is quite preponderant in this food displaying also a high sequence similarity (>52%) and identity (>38%) to several plant 2S albumins belonging to other nuts.

Data dependent proteomic analysis was performed on two different chocolate samples and all MS/MS data collected were processed via the commercial Proteome Discoverer software using SEQUEST as probability-score algorithm (1–3). This is one of the most popular scoring schemes, measuring the degree of correlation between the experimentally observed and the theoretical MS/MS spectra of peptides present in protein databases, and determines the peptide sequence yielding the best correlation score, or Xcorr. According to our results, the protein coverage was deemed satisfactory in most of the identifications, and the presence of unique peptides assigned to a single and specific protein confirmed the reliability of protein identification. For database searching a restriction to *Theobroma cacao* database was selected as preferential choice, and proteins such as 21 kDa seed protein OS (coverage = 30%), and Vicilin-A (coverage = 16.3%), putative OS, both belonging to *Theobroma cacao*, were identified and found common to both types of chocolate analyzed. For more details on protein identification see Supplementary Materials (Table S1). 

**Figure 3:**
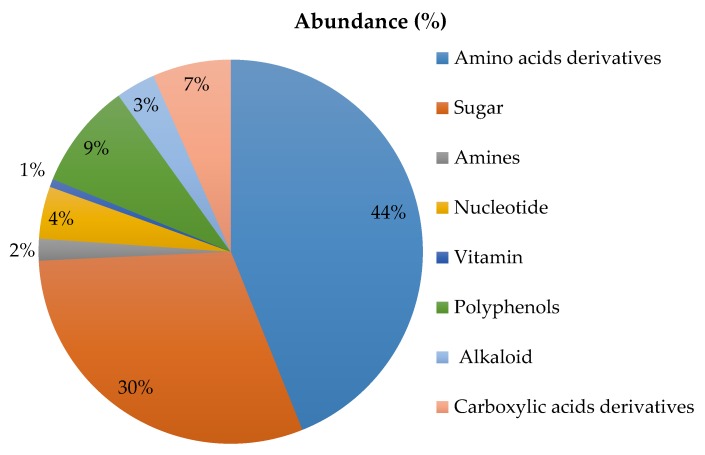
Mean abundance (%) of the chemical profiling in chocolates.

Figure 3 shows the principal classes of compounds identified in the two chocolates analysed by HPLC-MS/MS method. Data obtained from the data-dependent untargeted mass spectrometry analysis were processed via the commercial software Compound Discoverer leading to a total of 130 compounds identified. All of them were further grouped into 8 main classes, according to their common features. Our findings show that both chocolates do not differ for metabolite composition both from a qualitative and semi-quantitative point of view, as found by comparing peaks areas of the precursor ions falling into each individual group by untargeted mass spectrometry experiments.

Summarizing, the most representative classes found were amino acids and amino acids derivatives (44%), sugar (30%), polyphenols (9%) and carboxylic acids derivatives (6%). Nucleotides, alkaloids, amines and vitamins, were also identified but at a lower percentage (see Figure 3). More detailed information about peak areas calculated for each class of compounds in both types of chocolates (two lots analysed for each type for two technical replicates each) are reported in Table S2 of Supplementary Materials. As far as the fatty acids identification is concerned, chocolate was submitted to a dedicated extraction procedure (described in Section 2.6.1) and the final extract was injected into the HPLC-MS/MS system by operating in data dependent acquisition mode. In Table S3 of the Supporting Material, the list of main fatty acids identified in this matrix is reported, along with the percentage of peak areas calculated for each precursor ion detected and normalized on the ion current sum. As shown, the means obtained were not statistically different between both chocolates (according to the independent t-test performed).

### 5.2. Organoleptic Evaluation

**Figure 4:**
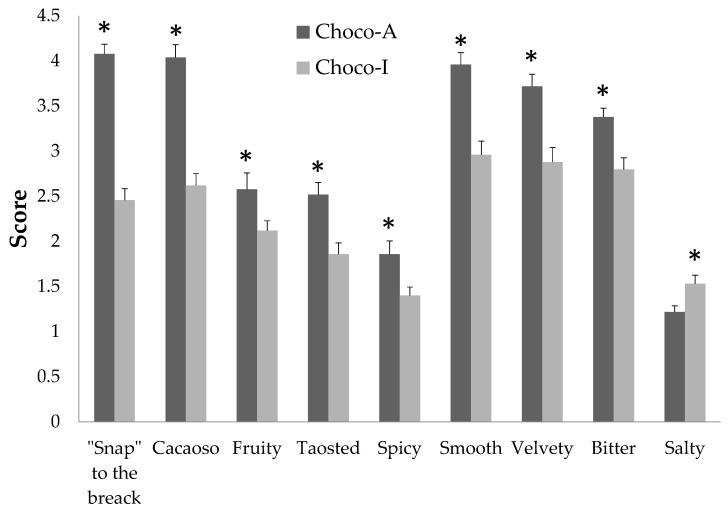
Score of sensory evaluation of two chocolates. Data are expressed as means ± SEM. * *p* < 0.05 Choco-A vs. Choco-I.

Scores about sensory evaluation for both chocolates are pictured in Figure 4, which shows the profile of scored domains with significant differences. Appearance as total and specific descriptors was comparable between Choco-A and Choco-I. The descriptors of “snap” on breaking, smell, texture (“smooth” and “velvety”) and taste (“bitter” and “salty”) in Choco-A, however, were significantly higher than the descriptors of Choco-I. 

### 5.3. Gastrointestinal Motility

**Figure 5:**
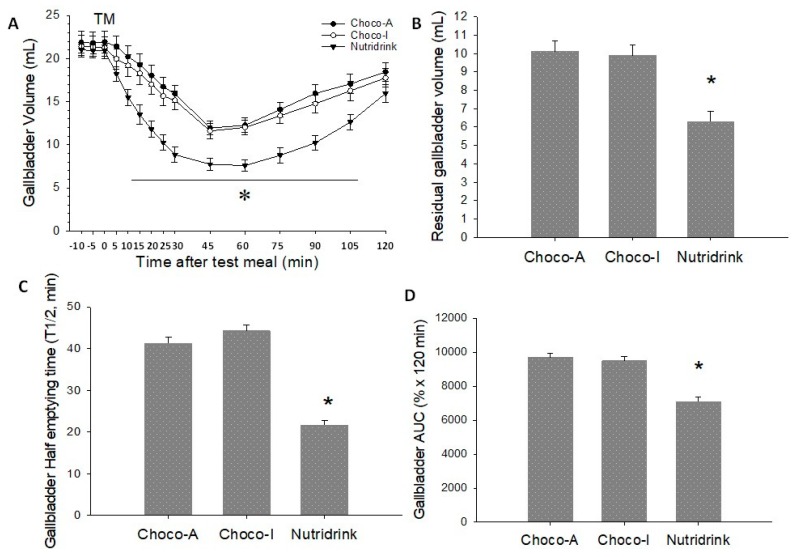
Gallbladder emptying parameters in response to ingestion of chocolates and test meal. (**A**) Time-dependent changes of gallbladder volume as mL; (**B**) Residual gallbladder volume as mL; (**C**) Half-emptying time as min; (**D**) Area under emptying curve (AUC). Data are expressed as means ± SEM. * *p* < 0.05 vs. Choco-A and Choco-I.

**Figure 6:**
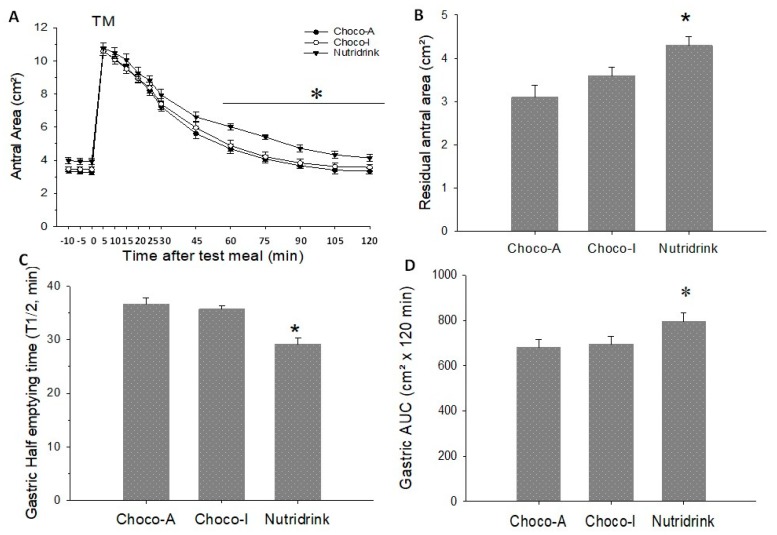
Gastric emptying parameters in response to ingestion of chocolates and test meal. (**A**) Time-dependent changes of antral area as cm²; (**B**) Residual antral area as cm²; (**C**) Half-emptying time as min; (**D**) Area under emptying curve (AUC). Data are expressed as means ± SEM. * *p* < 0.05 vs. Choco-A and Choco-I.

**Figure 7:**
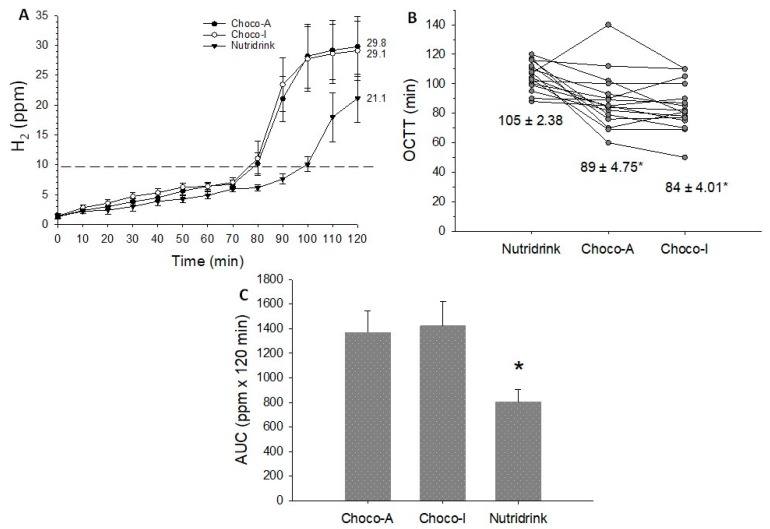
Orocecal transit/colonic fermentation parameters in response to ingestion of chocolates and test meal. (**A**) Time-dependent changes of H_2_ levels (ppm) in exhaled air; (**B**) Orocecal transit time (OCTT) as individual values and mean ± SEM reported below; (**C**) Area under H_2_ curve (AUC). Data are expressed as means ± SEM. * *p* < 0.05 vs. Choco-A and Choco-I.

The results of the functional gastrointestinal motility studies in response to chocolate meals and standard test meal (Nutridrink) appear in Figure 5, Figure 6 and Figure 7.

### 5.4. Gallbladder

Fasting gallbladder volumes at baseline were comparable between meals over three different days and ranged from 20.7–21.4 mL. All markers of gallbladder motility were comparable between Choco-A and Choco-I. In addition, the final refilling volume and the refilling time were comparable between the three meals. However, the ingestion of the two dark chocolates was associated with a greater residual volume, a smaller area under curve (AUC), and longer half-emptying time than Nutridrink. Specifically, the ingestion of each test meal induced a mean gallbladder response of 52%, 53%, and 70% in Choco-A, Choco-I, and Nutridrink, respectively. The graphic analyses of the emptying curves appear in Figure 5A–D, with respect to gallbladder volume, residual volume, half-emptying time, and AUC. Whereas the emptying curves with chocolates showed comparable profiles, emptying was faster and more complete with Nutridrink.

### 5.5. Stomach

On three different days, basal (fasting) antral areas were comparable between the three meals and ranged from 3.3–3.9 cm². The ingestion of the three meals induced comparable antral dilatation. The residual antral area and the AUC in cm^2^ were significantly larger with Nutridrink, but this difference was lost with AUC as percentage. Half-emptying time was significantly shorter with Nutridrink than chocolate. The graphic analysis of the emptying curves appears in Figure 6A–D. For in depth information please check Table S4 in Supplementary Materials. 

### 5.6. Small Intestine

Data concerning the orocecal transit time (OCTT) appear in Table S4 and Figure 7A–C. The ingestion of each test meal caused a significant increase in H_2_ levels in exhaled air, greater in chocolates than Nutridrink (Figure 7A). OCTT was shorter and AUC (ppm x 120 min) greater with chocolates than Nutridrink (Table S4).

Notably, during the time of the tests, the kinetics of motility (stomach, gallbladder and small intestine) appeared complete (i.e., emptying/refilling of the gallbladder; filling/emptying of the stomach; consistent increase of H_2_ as a marker of small intestinal transit due to lactulose fermentation). Thus, results appeared to be very similar between both types of chocolate whereas were highly different when compared with the Nutridrink test meal.

## 6. Discussion

In this comprehensive study, we combined chemical analyses, organoleptic evaluation, and gastrointestinal motility studies of two types of dark chocolates, while providing novel and physiologically relevant updates.

According to the literature [4,32,33], we confirm the high levels of polyphenol contained in both chocolates analysed, with particular regard to theobromine, procyanidin and epicatechin. Other compounds detected by mass spectrometry untargeted analysis are catechins, quercetin and chlorogenic acid considered phenols endowed with antioxidant properties. The wealth of polyphenol and the associated beneficial effects in relation to their antioxidant and anti-inflammatory properties, place chocolate among the nutraceuticals, encouraging its consumption in the diet of healthy and dismetabolic individuals [6,34].

Regarding other chemical analysis, the category encompassing amino acids and their derivatives, accounts for the total compounds detected by MS/MS analysis. The levels of amino acids detected are in correlation with the protocol used for chocolate production. It has been reported that cacao beans fermented for 120 h (control (TI)) showed higher amino acid content, specifically for aspartic acid, glutamic acid, alanine, isoleucine, proline and valine (hydrophobic). In valine, we also detected high levels of aspartic acid and glutamic acid as well as phenilalanine followed by other amino acids at a lower level [35]. Phenylalanine is in particular included among the essential amino acids, namely those molecules the body is not able to produce on its own. These amino acids are crucial for the production of tyrosine, but also for the production of epinephrine/norepinephrine and dopamine involved in the body’s normal functioning, including mood and stress responses.

As far as the lipid profile is concerning the class of FA, in particular, plays an important role in the beneficial effects exerted on different organs, especially with regard to the essential FA represented by the polyunsaturated FA. Cocoa butter typically contains both monounsaturated and saturated FA. Palmitic acid and stearic acid (C*_16:0_*, C*_18:0_*) are the most abundant saturated FA while oleic acid and linoleic acid (C*_18:1_* and C*_18:2_*), are the most representative of the monounsaturated and polyunsaturated FA [36,37]. According to our results the FA identified in the chocolate analysed are: stearic acid representing the most abundant (~28%), followed by the polyunsaturated FA linoleic acid also called omega-3 (~20%), myristic acid (19%) and oleic acid (16%). Lower percentages of lauric acid, palmitoleic acid, pinolenic acid, arachidic acid and pentadecanoic acid were also calculated in the extracts accounting respectively for 6%, 3%, 3%, 2% and 1%. In particular the high content of stearic acid is important from a clinical perspective since it has been reported to be anti-atherogenic contributing to reducing the risk of cardiovascular disease [38]. Alpha-linolenic acid, preponderant in chocolate, is popular for preventing and treating diseases of the heart and blood vessels enhancing the beneficial effect exerted by this food against heart attack and high blood pressure, lowering cholesterol in the blood [39].

In addition to the health promoting properties of flavonols and unsaturated fatty acids identified, dark chocolate 70% contains an average of 8 g of fiber per 100 g corresponding to 32% of the fiber recommended daily allowance for a mature adult. Fibers play a pivotal role in promoting health, especially because they are reported to improve the low density lipoprotein (LDL): high density lipoprotein (HDL) ratio, and also reduce the risk of type 2 diabetes [40,41]. In particular cocoa husks are a particular good source of dietary fiber, mostly insoluble fiber [42]. Such a product might be of interest for the food industry, with potential use as an ingredient in fiber-rich functional foods. Besides the benefits derived from its high fiber content, cocoa fibers would provide protection against oxidative damage by means of its content in phenolic compounds (epicatechin) which are absorbed, maintaining the antioxidant properties in vivo.

### 6.1. Perception Study

Results confirm that in the fasting state volunteers can appreciate several differences between the two dark chocolates. Most evident differences concerned significantly increased scores for domains like “snap”, smell, texture and bitter taste, in Choco-A compared with Choco-I. Differences in both domains are likely to be dependent on different texture composition of artisanal chocolate vs. industrial chocolate. In principle, such differences might partly influence the early kinetic response of the gastrointestinal tract due, for example, to the neural cephalic phase anticipating the neuro-hormonal gallbladder contraction in response to fat. Upon meal ingestion and arrival in the gastro-duodenal segment, in fact, both neural (vagal), and hormonal (cholecystokinin, CCK) stimuli will govern the subsequent gastro-cholecysto-intestinal motility [43,44,45].

### 6.2. Motility Study

We further explored the effects of chocolates on gastrointestinal motility. This aspect has never been properly assessed before, and in this study it was performed in correlation with a comprehensive evaluation of sensory properties. The two chocolates were fully comparable in terms of lipids, protein and carbohydrates content, whereas the test meal had the same amount of fat but a greater amount of proteins and carbohydrates than both chocolates. The final volume of the lactulose containing the three meals, however, was fully comparable to achieve similar initial gastric response. 

In the gallbladder, both chocolates induced comparable emptying responses by achieving more than 50% gallbladder emptying by 40 min. This prokinetic response provided adequate flushing of gallbladder bile and might be also useful to prevent gallbladder stasis in a number of conditions (i.e., obesity, insulin resistance, diabetes mellitus, etc.) [46], predisposing to super saturation of gallbladder bile, a condition at risk of cholesterol accumulation and gallstone growth [43].

In the stomach, both chocolates induced comparable emptying profiles with maximal antral area comparable to that obtained with Nutridrink (i.e., ~12 cm^2^). This response likely depends on the effect of three isovolumetric meals. Gastric emptying speed, however, was faster than gallbladder emptying time. By 50% of refilling time (79 min), both chocolates produced a significant increase of breath hydrogen. Approximately 10 min after 50% refilling time, the orocecal transit time was observed. Of note, both chocolate meals produced similar transit time. By contrast, the test meal was faster in inducing gastric emptying and gallbladder emptying, but slower with gallbladder refilling and orocecal transit time. These findings might easily be explained by different characteristics of the meals and by the more intense test meal-depending gallbladder contraction, possibly modulating both refilling and subsequent intestinal transit [47,48].

We must point out that none of the subjects reported symptoms related to dyspepsia, i.e., bloating, nausea and epigastric pain. We confirm that the mean score of dyspepsia is extremely low in healthy subjects, as shown in previous studies [27,49,50,51]. The ingestion of the test meals was not associated with dyspeptic symptoms (especially fullness) throughout the observation time. Likely, the moderate volume (215 mL) and the isosmotic/fat composition of the test meals (12 g) fat, was not sufficient to trigger upper gastrointestinal symptoms in these groups of subjects.

This study provides a great deal of relevant information when dealing with nutrients and type of foods, and both the composition of food and the pathophysiological behavior of the gastrointestinal motility with respect to organoleptic features are important. Here, we show that the fat content and final volume of liquid meals were comparable, but profound differences exist in terms of gastric, gallbladder and intestinal transit. 

In conclusion, chocolate as a dark food, also introduced as a gratifying food, in its artisanal form has characteristics that suggest a favorable use compared to industrial chocolate. The artisanal chocolate has good kinetic properties, comparable to other chocolates (industrial chocolate), but has better organoleptic characteristics. Therefore, the ingestion of a small amount of chocolate equivalent to a discreet contraction of the gallbladder is able to stimulate the gallbladder, induce a contraction of the stomach and also speed up the transit time that proved to be faster than a standard meal with a good fermentation curve.

Studies are in progress to evaluate other organoleptic aspects and kinetic aspects, for example on gastrointestinal permeability, to assess the impact of dark chocolate on lipid metabolism. Studies are also in progress to assess chronic effect following the intake of chocolate. 

## Figures and Tables

**Table 1:** Clinical characteristics of enrolled subjects.

	Organoleptic Evaluation	Gastrointestinal Motility Studies
Number	50	16
Males:Females	25:25	8:8
Age, yrs (Range)	28 ± 0.9 (21–57)	25 ± 0.6 (21–29)
BMI, Kg/m² (Range)	22.3 ± 0.3 (18.6–31.0)	21.9 ± 0.4 (18.8–25.1)

Legend: BMI, Body Mass Index; Data are mean ± SEM.

**Table 2:** Composition of meals.

	Choco-A	Choco-I	Nutridrink
Chocolate (g)	28.6	27.3	-
Water (mL)	175	176	200
Lactulose (mL)	15	15	15
Final Volume (mL)	215	215	215
Fat g (%)	12 (43)	12 (45)	12 (20)
Protein g (%)	2.50 (9)	2.70 (10)	12 (20)
Carbohydrates g (%)	10.50 (38)	9.30 (35)	36.80 (60)
Soluble fiber g (%)	2.69 (10)	2.73 (10)	-
Salt g (%)	-	0.07 (0)	-
Kcal (kJ)	165 (684)	161 (668)	300 (1260)
Density (g/mL)	1.02	1.02	1.09

**Table 3:** Composition of chocolates.

	Choco-A	Choco-I	*p* Value
Carbohydrates (%)	45.4 ± 7.78	45.1 ± 7.00	0.56
Soluble fiber (%)	5.1 ± 0.78	4.9 ± 1.69	0.95
Fat (%)	41.2 ± 0.99	41.0 ± 1.41	0.63
Protein (%)	8.3 ± 0.57	8.9 ± 0.35	0.17
Moisture (%)	1.4 ± 0.05	1.5 ± 0.03	0.08
Ash (%)	1.7 ± 0.04	1.8 ± 0.06	0.29

Data are mean ± standard deviation (SD). Comparisons between groups performed by Independent T-test. Abbreviations: Choco-A, artisanal chocolate; Choco-I, industrial chocolate. The values are the mean of two different samples.

**Table 4:** Chemical analyses of chocolates.

	Choco-A	Choco-I	*p* Value
Total phenol content (TPC) (mg GAE/g)	20.66 ± 1.28	21.53± 2.14	0.57
2,2-Azino-bis(3-ethylbenzothiazoline-6-sulphonic acid (ABTS) (µmol TE/g)	98.39 ± 1.70	88.99 ± 4.20	0.02
2,2-diphenyl-1-picrylhydrazyl (DPPH) (µmol TE/g)	79.81 ± 5.4	68.65 ± 1.39	0.02
Theobromine (µg/g)	1238.68 ± 73.63	1436.61 ± 62.64	0.02
Catechin (µg/g)	24.00 ± 4.78	21.63 ± 1.98	0.47
Procyanidin B2 (µg/g)	72.95 ± 1.70	71.28 ± 4.23	0.62
Epicatechin (µg/g)	112.10 ± 10.83	101.44 ± 8.25	0.24

Total phenol content, antioxidant activity evaluation and determination of the main phenolic compounds and alkaloids by HPLC-DAD. Data are mean ± standard deviation (SD). Comparisons between groups performed by Independent T-test. GAE: Gallic acid equivalents; TE: Trolox equivalents. The values are the mean of two different samples.

## Supplementary Materials

The following are available online at https://www.mdpi.com/2072-6643/12/4/939/s1.
