# Supplementary material for: Chemical Characterization, Gastrointestinal Motility and Sensory Evaluation of Dark Chocolate: A Nutraceutical Boosting Consumers’ Health
Source: Nutrients. 2020 Mar 28;12(4):939. doi: 10.3390/nu12040939 (PMC7230710; doi:10.3390/nu12040939)
Supplement: Supplementary file 1 [file nutrients-12-00939-s001.pdf]

Table S1. Mean score retrieved by the software by searching into Theobroma Cacao database proteotypic peptides of Choco-A and Choco-I extracts.

Accession	Description	Coverage	Peptides	PSMs	Unique Peptides	Protein Groups	AAs	MW [kDa]	Calc. pI	Score Sequest HT
P32765	21 kDa seed protein OS=Theobroma cacao	30,3	13	23	11	1	221	24,0	6,1	16,6
Confidence	Sequence	Modifications	Protein Groups	Proteins	PSMs	Master Protein Accessions	Missed Cleavages	Theo. MH+ [Da]	XCorr Sequest HT	Confidence Sequest HT
High	HSDDDGQIR		1	2	2	P32765	0	1042,455	2,78	High
High	AGGGGL		1	19	4	P32765	0	431,2249	2,16	High
High	GAGGGGI		1	9	4	P32765	0	488,2463	1,56	High
High	TDVN		3	79	1	P32765; A0A061EXD4; A0A061FZ39	0	448,2038	1,25	High
High	KAGVI		1	66	2	P32765	0	487,3239	1,22	High
High	VSTDVNIEFVPIR		1	3	2	P32765	0	1488,806	2,49	High
High	SDLDNGTPVIFSNADSK	1xDeamidated_modificato [T/N/L/S]	1	2	7	P32765	0	1780,824	2,02	High
High	ANSPV		1	7	1	P32765	0	487,2511	1,53	High
Medium	VPLR		1	382	4	P32765	0	484,3242	0,97	Medium
Medium	SSISGAGGGGLALGR		1	2	2	P32765	0	1259,67	1,68	Medium
Medium	DVVR		2	262	2	A0A061E098; P32765	0	488,2827	1,1	Medium
Medium	VPIR		1	382	4	P32765	0	484,3242	1,04	Medium
Medium	DSKDDVVR		1	3	1	P32765	0	933,4636	1	Medium
A0A061EM85	Vicilin-A, putative OS=Theobroma cacao OX=3641 GN=TCM_020665 PE=4 SV=1	16,3	10	20	9	1	566	65,5	6,6	5,8
Confidence	Sequence	Modifications	Protein Groups	Proteins	PSMs	Master Protein Accessions	Missed Cleavages	Theo. MH+ [Da]	XCorr Sequest HT	Confidence Sequest HT
High	QQEEELQR		1	2	4	A0A061EM85	0	1059,507	2,17	High
High	QQEEELQR	1xDeamidated_modificato [Q]	1	2	4	A0A061EM85	0	1060,491	2,22	High
High	KDQPI		1	8	4	A0A061EM85	0	600,3352	1,42	High
Medium	GTVVSVPAGS		1	2	2	A0A061E	0	2264,14	1,66	Medium

	TVYVVSQDNQEK				M85				
Medium	KIVD	2	734	4	A0A061DLZ2; A0A061EM85	0	474,2922	1,12	Medium
Medium	FRDEEGNF	1	2	2	A0A061EM85	0	1013,432	1,5	Medium
Medium	APLSPGDVFVAPAGH	1	2	2	A0A061EM85	0	1434,738	1,48	Medium
Medium	ESYNVQR	1	2	1	A0A061EM85	0	895,4268	1,27	Medium
Medium	ELSFGVPSK	1	1	1	A0A061EM85	0	963,5146	1,1	Medium
Medium	APLSPGDVF	1	2	1	A0A061EM85	0	902,4618	0,93	Medium

Tab. S2. Average of peak area ± the respective standard deviation (s.d.) detected in Choco A and Choco I for each class of compounds identified by untargeted LC-MS/MS analysis and software processing.

Compound class	Averaged peak area Choco A (±s.d.)	Averaged peak area Choco I (±s.d.)
Aminoacid derivatives	37038894 ± 3635074	29985489 ± 2476873
Sugar	29297484 ± 14143770	19942092 ± 7661307
Amines	1705278 ± 347599	888268 ± 201120
Nucleotide	4216869 ± 869526	2990453 ± 400958
Vitamin	546492 ± 147690	355318 ± 73624
Alkaloid	2525364 ± 340321	2282593 ± 371813
Polyphenols	7730866 ± 1513332	5044820 ± 1080927
Carboxylic acids derivatives	5096328 ± 980892	3543273 ± 577440

Table S3. Peak areas obtained by peak integration along the total ion chromatogram referred to fatty acids detected in Choco-A and Choco-I.

	Elemental composition	Peak area Choco-A	Peak area Choco-I	% of area Choco-A	% of area Choco-I	P value
**Stearic acid**	$C_{18}H_{36}O_2$	4518302.43 ±737307	4538221.80 ±694012	27.7	28.9	0.99
**Arachidic acid**	$C_{20}H_{40}O_2$	341755.12 ±38212	329632.69 ±55104	2.1	2.1	0.89
**Palmitoleic acid**	$C_{16}H_{30}O_2$	485937.16 ±40134	519719.16 ±14817	3.0	3.3	0.54
**Linoleic acid**	$C_{18}H_{32}O_2$	2972868.67 ±1901117	3490782.81 ±22323	18.2	22.2	0.77
**Pentadecanoic acid**	$C_{15}H_{30}O_2$	156393.27 ±42860	191022.78 ±10108	1.0	1.2	0.38
**Myristic acid**	$C_{14}H_{28}O_2$	3237288.50 ±3237289	3005033.72 ±3005034	19.8	19.1	0.69
**Oleic acid**	$C_{18}H_{34}O_2$	3117859.06 ±3418503	2232287.53 ±2232288	19.1	14.2	0.25
**Pinolenic acid**	$C_{18}H_{30}O_2$	452454.84 ±452454	391391.78 ±391391	2.8	2.5	0.07
**Lauric acid**	$C_{12}H_{24}O_2$	1049579.00 ±1049579	1018182.25 ±1018182	6.4	6.5	0.86

Data are expressed as means± standard deviation.

Table S4. Gastrointestinal motility studies in response to liquid test meal and two chocolate test meals in 16 healthy subjects.

	**Choco-A**	**Choco-I**	**Nutridrink**	**P value**
Gallbladder (GB)				
Fasting vol., mL	21.4±1.27	21.3±1.24	20.7±0.68	0.79
(median)	(21.7)	(20.8)	(21)	
Residual vol., mL	10.1±0.56[a]	9.9±0.57[a]	6.4±0.57[b]	0.00
(median)	(10.5)	(10.1)	(7.6)	
Residual vol., %	47.6±1.91[a]	46.7±1.93[a]	29.4±2.12[b]	0.00
(median)	(46.4)	(47.5)	(30.4)	
Time to residual volume, min	56.3±3.99	55.3±4.04	51.3±3.02	0.57
(median)	(50)	(45)	(45)	
Final refilling volume, mL	18.4±0.98	17.8±1.09	15.9±1.06	0.15
(median)	(19)	(18.9)	(17)	
Final refilling volume, %	84.7±2.86	82.3±3.26	76.2±3.54	0.11
(median)	(84.4)	(83.8)	(79.8)	
AUC, mL × 120 min	1900.4±99.85[a]	1808.6±103.32[a]	1310.9±82.02[b]	0.00
(median)	(1815.1)	(1771.2)	(1377.6)	
AUC, % × 120 min	8694.8±234.58[a]	8487.9±252.74[a]	6090.9±261.91[b]	0.00
(median)	(8234.1)	(8261.3)	(6289.5)	
Half-emptying time, min	41.3±1.46[a]	44.2±1.36[a]	21.7±1.06[b]	0.00
(median)	(42.3)	(42.8)	(21.1)	
Half-refilling time, min	78.9±3.80	83.6±4.38	89.4±4.70	0.31

(median)	(76.5)	(84.5)	(92)	
Stomach				
Basal antral area, cm^2	3.3±0.12[a]	3.4±0.14[a]	3.9±0.20[b]	0.01
(median)	(3.4)	(3.4)	(3.9)	
Max postprandial antral area, cm^2	10.6±0.21	10.6±0.27	10.7±0.30	0.78
(median)	(10.3)	(10.4)	(10.5)	
Residual antral area, cm^2	3.1±0.27[a]	3.6±0.20[a]	4.3±0.20[b]	0.00
(median)	(3.4)	(3.4)	(4.7)	
AUC, cm^2 × 120 min	664.9±22.99[a]	684.5±28.09[a]	773.7±25.17[b]	0.01
(median)	(644.5)	(652.1)	(755.3)	
AUC, % × 120 min	3629.0±161.11	3624.6±195.50	3599.5±241.51	0.99
(median)	(3507.6)	(3475.6)	(3489.0)	
Half-emptying time, min	36.6±1.24[a]	35.7±0.69[a]	29.1±1.15[b]	0.00
(median)	(35)	(35)	(29.7)	
Small intestine				
Orocecal transit time, min	89±4.75[a]	84.3±4.01[a]	105.0±2.38[b]	0.01
(median)	(85.0)	(81.5)	(105.5)	
AUC, ppm × 120 min	1367.8±177.07	1421.9±200.03	805.9±99.63	0.05
(median)	(1290.0)	(1245.0)	(665.0)	

Each test meal randomly given to the same subject. AUC, area under curve; data are expressed as means± standard error and (median); Comparisons between groups performed by ANOVA followed by Tukey-Kramer's post-hoc test; different letters indicate significant differences.
